# Mockup Test of UHPFRC Prestressed Arch

**DOI:** 10.3390/ma19153193

**Published:** 2026-07-27

**Authors:** Martin Válek, Šárka Pešková, Jiří Litoš, Marcel Jogl, Eva Horáková, Michal Mára, Pavel Horák, Petr Konvalinka, Petr Vítek, Jan Valentin

**Affiliations:** 1 Faculty of Civil Engineering, Czech Technical University in Prague, Thákurova 7, 166 29 Praha, Czech Republic; litos@fsv.cvut.cz (J.L.); marcel.jogl@fsv.cvut.cz (M.J.); michal.mara@fsv.cvut.cz (M.M.);; 2 Hochtief CZ a.s., Plzeňská 3217/16, 150 00 Praha, Czech Republic; petr.vitek@hochtief.cz (P.V.);

**Keywords:** arch, UHPFRC, cracking, failure, model, mockup

## Abstract

A two-hinged arch is part of a traditional structural element that can last for thousands of years. This mockup experiment tested two prestressed UHPFRC (Ultra-High-Performance Fibre-Reinforced Concrete) arches with a span of 10.32 m up to failure. Each arch was composed of two semi-arches, which were joined and slightly prestressed to ensure watertightness in the joint. The load acted on one third of the span, inducing eigenshape-like displacements. The first tensile crack appeared under the load at 60 kN, followed by shear cracks and tensile failure in an approximately opposite cross-section. The maximum load reached 170 kN with brittle failure afterwards. The behavior was successfully validated using a 3D finite element analysis and damage-plasticity material model. The mockup experiment proved that upscaling to a real ecoduct arch becomes possible, creating a unique structure with a very long service life.

## 1. Introduction

**Figure 1:**
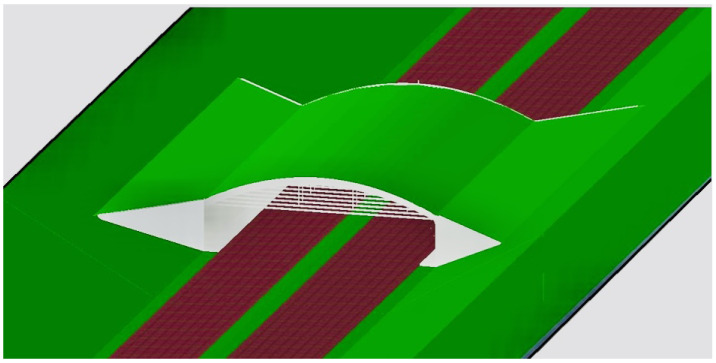
3D model of the ecoduct.

Although UHPFRC has been successfully applied in several bridge structures over the past two decades, existing research focuses almost exclusively on conventional bridge systems, i.e., structural members designed for traffic loading, dynamic effects, and the standard limit states of bridge engineering. In contrast, the available literature does not provide experimental verification of UHPFRC structural systems specifically developed for wildlife overpasses (ecoducts), whose loading regime, geometry, and operational conditions differ fundamentally from those of bridges. An ecoduct represents a structural typology positioned between a bridge and a shallow tunnel: it is extremely wide, subjected to long-term loading from deep soil cover, and experiences negligible live load from traffic. These characteristics generate a distinct stress–strain environment, particularly in the arch, contact joints, and prestressed regions, which cannot be extrapolated from existing UHPFRC bridge studies. Furthermore, none of the published research addresses a self anchored UHPFRC arch with a monolithic contact joint and bidirectional prestressing, which is a structural concept developed specifically for ecoduct applications and has never been experimentally validated. Therefore, the present study provides the first physical verification of this structural system, including an investigation of the failure mechanism up to complete collapse—an area in which numerical models are known to exhibit limited reliability. By experimentally capturing the nonlinear and post-peak behavior of a large-scale UHPFRC arch element under loading conditions representative of ecoducts, this work fills a significant gap in the current state of knowledge and offers new insights into the behavior of large UHPFRC arch components subjected to soil dominated loading scenarios. The aim of this mock-up experiment is to verify the feasibility and suitability of the proposed structural system for the construction of ecoducts. An ecoduct structure consists of a self-anchored arch with a bridge deck assembled from two parts connected at a contact joint. The joint between the beams will also be monolithically cast using UHPFRC, and the beams will be prestressed in both the longitudinal and transverse directions to ensure the watertightness of the monolithic joint. The 3D model of the ecoduct is shown in Figure 1.

**Figure 2:**
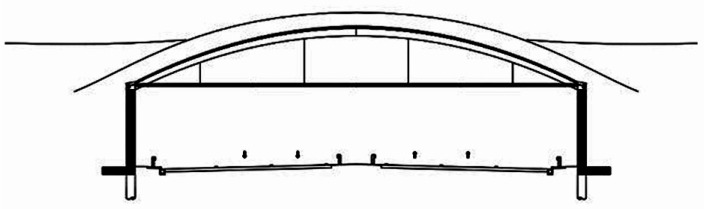
Cross-section of the ecoduct structure.

It is assumed that the highway, with up to 3 + 3 lanes, will be bridged without a central pillar in the central dividing strip. The span will be from 27.5 m, for 2 + 2 lines, to 40 m. The individual beams are coupled with a thin monolithic plate; above it is a layer of soil for vegetation growth. See Figure 2.

The horizontal forces at the ends of the arch will be captured by prestressed cables suspended under the arch. The arch then acts on the foundations only with vertical forces, and can also be founded on micropiles. In the case of exceptionally suitable geological conditions, the tie rod can be omitted and the forces transferred directly to the foundations.

**Figure 3:**
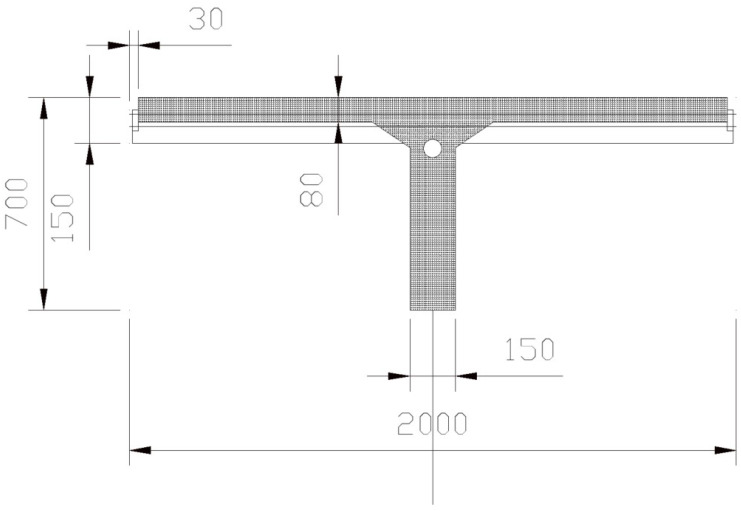
Cross-section of one prefabricated segment.

It is assumed that the ecoduct will be assembled on site from individual prefabricated segments. The arch has a T-shaped cross-section and consists of two parts connected in the middle by a monolithic contact joint. The width of the beam is two meters, and the height is 0.7 m. The thickness of the plate is 80 mm and the width of the web is 150 mm; see Figure 3. In the longitudinal direction, the beam is prestressed at the center of gravity with two to eight cables of the Monostrand prestressing system. At the arch’s toe, its ends are subsequently connected by a tie rod.

The structure is also suitable for construction over an operating highway with minimal closures gradually in one lane. In the case of highway modernization, construction can be coordinated with the closures of individual lanes.

The arched beam was chosen for a number of advantages that this concept brings. With a suitably designed curvature, the entire cross-section is stressed by pressure, which is very advantageous for building materials with high compressive strength and low tensile strength, such as concrete. The risk of tensile cracks is eliminated, which increases both the waterproofing and the service life of the structure. Together with the use of UHPFRC, it allows waterproofing to be omitted. This is often the weak point of structures, as its service life is often an order of magnitude shorter than the service life of the structure. It is subject to failure, requiring expensive repairs and sometimes even shortening the service life of the structure.

Significant examples of structures of this type in the world are Sunyudo Footbridge in South Korea, built in 2002; Fuzhou University Landscape Bridge in China, built in 2015; Baquitan Bridge, also in China; and the Wild Bridge in Austria, built in 2010 [1]. Sunyudo Footbridge is a prefabricated segmental arch made of UHPFRC with a span of 120m. It is a π-girder. The arch width is 4.3 m and the depth is 1.3 m [2]. The Baqiutian Bridge is designed with the concept of sustainability. The bridge is a deck arch with reinforced double UHPFRC ribs. The arch rib has an effective span of 34 m, is prefabricated in three segments and is connected by dry joints, which can reduce the shrinkage stress of the UHPFRC structure and improve construction efficiency. For the bridge, the deck structure with the bridging element is directly connected to both spans of the approach, forming a continuous structure that is extended to the approach plate behind the abutment, thus eliminating all deck expansion joints in the entire bridge and becoming a jointless bridge. The bridge thus does not encounter the same problems as conventional articulated bridges during operation, i.e., damage, maintenance and replacement of bridge deck expansion joints, as well as their negative impact on traffic and structures [3]. Road Bridge–Wild is a pilot project for an arch bridge made of UHPFRC in Austria. The 157 m long bridge consists of two fore-span bridges and a 70 m long UHPFRC segmental arch [4].

UHPFRC is a relatively new generation of cement material with very high strength, ductility and durability. It can be considered as a combination of three concrete technologies: self-compacting concrete (SCC), fiber-reinforced concrete (FRC) and high-performance concrete (HPC). The French Interim Recommendation (AFGC 2002) [5] defined UHPFRC as concrete with a characteristic compressive strength of at least 150 MPa using steel fiber reinforcement to ensure tensile behavior. However, compressive strengths in excess of 200 MPa and flexural tensile strengths in excess of 15 MPa can be achieved. The technology for producing UHPFRC is based on four basic principles:

- Reducing the water/binder ratio and minimizing the porosity of the composite by optimizing the granular mix through a wide range of powder size classes.
- Improving the microstructure by post-solidification heat treatment to accelerate the pozzolanic reaction and increase mechanical properties.
- Increasing homogeneity by removing coarse aggregate, which leads to a reduction in the mechanical effects of heterogeneity.
- Improving ductility by including a sufficient volume fraction of small steel fibers, which also improves the tensile strength of concrete [6].

The UHPFRC material is relatively new, but has already been successfully used for bridge construction in many countries including Australia, Austria, Canada, China, Czech Republic, France, Germany, Italy, Japan, Malaysia, Netherlands, New Zealand, Slovenia, South Korea, Switzerland and the United States. Most projects in the mentioned countries have been motivated by government agencies as initial demonstration projects intended to encourage further implementation [7]. In the Czech Republic, these are mainly pedestrian bridges, such as the footbridge over the Lubina river in the city Příbor, the pedestrian and cycle bridge in the vicinity of the Black Bridges in the city of Tábor, and the footbridge over Dřetovice stream in Vrapice—a district of Kladno city [8]. However, Ekodukt represents the application of UHPFRC on a completely new type construction. Although an ecoduct is defined as a special bridge object that overcomes an artificial linear obstacle, there are significant differences compared to a classic bridge, both in geometry and in load. It turns out that one of the important parameters of an ecoduct is its width. The wider, the better. Today, 50 m is considered the minimum good width, but there are also ecoducts that are hundreds of meters wide. However, this changes the character of the structure from a bridge to a tunnel. The same is true with the load. In Central Europe, crossing animals do not generate any significant load on the structure. It is loaded with filled soil, but it is necessary to count on the occasional movement of heavy equipment during maintenance, and also the possibility of removing part of the soil. Therefore, the load here is closer to the load of a tunnel than to a bridge. Since it can be assumed that for most of the operating time of the structure, the main load on the structure will be the load from its own weight and from the filled soil, the most suitable shape is an arched beam, where with a suitable geometry we can achieve the compression of the entire cross-section. The requirement for self-anchoring, which is solved by adding a tie rod, is based on the fact that in the case of building an ecoduct in a plane, it is not necessary to solve the complex problem of transferring horizontal forces to the subsoil. UHPFRC was chosen as the most suitable material for the production of the beam for economic and environmental reasons. These are mainly:

- Savings in material, where concrete with better properties allows the use of much more subtle elements for the structure. This allows up to 50% savings in material compared to conventional concrete structures.
- Savings in maintenance costs. UHPFRC is a waterproof non-absorbent material with closed pores, which allows it to resist both weather conditions and the growth of plant roots.
- Long service life of the structure, as the expected service life of UHPFRC is significantly higher than the service life of structures made from other building materials. The expected service life of a structure made of UHPFRC is at least 100 years.
- UHPFRC is waterproof, so the structure can be simplified and difficult waterproofing solutions are no longer needed.
- Time savings in construction, where structural elements can be manufactured in advance and imported to the construction site due to their lighter weight.
- Part of the cement in UHPFRC can be replaced with secondary materials.
- Last but not least, an advantage is the possibility of construction even over existing roads with minimal traffic restrictions.

An environmental impact assessment and life cycle cost analysis, which provide insight into the wider implications of using UHPFRC, is presented in [9]. The environmental aspects are illustrated by the determination of ${\mathrm{CO}}_{2}$ emissions for several cases. The relatively large dispersion of ${\mathrm{CO}}_{2}$ emission values indicates the need to address the optimization of the mix composition from an environmental perspective.

The authors of a study comparing patented and unpatented high-strength concrete mixtures in terms of energy consumption, ${\mathrm{CO}}_{2}$ emissions and costs in [10] came to a rather surprising conclusion. While patented UHPFRC mixtures usually showed higher compressive strength, they were also associated with higher energy consumption, emissions and costs. On the other hand, non-patented mixtures achieved comparable mechanical performance with a lower overall environmental and economic burden.

Further savings and ecological improvements can be obtained, for example, by mixing fly ash from municipal solid waste incineration [11]. The authors concluded that the use of MSWIFA as a cementitious material in UHPFRC offers a viable alternative to conventional cement, meeting the performance requirements of concrete while delivering environmental benefits and cost savings. This increases resource efficiency, reduces the environmental impacts of cement production, and supports the sustainable development of UHPFRC.

Compared to the huge number of structures made of conventional concrete, UHPFRC has so far only been used in a small number of buildings. Therefore, one cannot fully rely on the results of numerical modeling and it is necessary to verify the load-bearing capacity and, above all, the possible nature of the failure of the main arch beam by experiment—especially in the subsequent stages of failure after reaching maximum load-bearing capacity up to the total collapse of the structure. In these phases, the accuracy of the numerical model decreases and its results cannot be relied upon too much.

## 2. State of the Art

In [12], the mechanical properties of UHPFRC T-section beams were investigated on 1.5 m long specimens with varying volume fraction of steel fibers. In addition to steel fibers, the specimens are reinforced with longitudinal reinforcement at the bottom edge. Furthermore, the influence of shear span ratio, longitudinal reinforcement ratio, and stirrup ratio on the bending and shear behavior of T-section beams was investigated. The test results showed that the use of steel fibers can significantly improve the shear capacity and can change the failure mode. If the steel fiber content is <2%, the specimens fail in shear, while if the content is higher, they fail in bending.

Similar conclusions were reached by the authors in the [13] study, which experimentally verifies the influence of fiber volume fraction, shear span to depth ratio, and compressive strength of matrix, on the bending strength of a rectangular beam. It was found that the greatest influence on the ultimate strength is the volume fraction of fibers and, as in the previous study, they consider 2% volume fraction of steel fibers as the limit where the failure mode changes.

The behavior of prestressed T-beams made of UHPFRC under bending load is investigated in [14]. The study confirms the feasibility and possible increase in structural stiffness by prestressing thin-walled FRP reinforced beams with a wall thickness of only 40 to 50 mm. The negative effect of small wall thicknesses is manifested in the earlier occurrence of splitting cracks. However, it does not affect the overall strength.

The results from experiments with UHPFRC beams without stirrups are then summarized in the Huang Yao et al. study [15], where test data from 487 beams were collected and an experimental database was created. Four different shear strength calculation models for UHPFRC beams were investigated in the study. These models were created from national specifications. The results indicate that although the standard equation is useful for predicting the shear capacity of UHPFRC beams, it consistently underestimates the actual values with an average ratio of experimental to calculated values above 1.5.

All of the above experiments were performed on specimens an order of magnitude smaller than the specimen used in this work. Experiments on structures of comparable dimensions were published in 2005 by Graybeal [16], who performed full-scale tests on AASHTO Type II beams manufactured without mild steel reinforcement and demonstrated that the inherent fiber reinforcement of UHPFRC could adequately serve in highway bridge girders. The beams are 80 ft long. This study also includes extensive material characterization that led to proposed design principles for UHPFRC structural members.

Another experimental investigation of the flexural behavior of a 30 m long prestressed UHPFRC-NC composite beam was published by Zhou et al. [17]. This is a beam consisting of a normal concrete deck coupled with a precast prestressed UHPFRC chamber. The experiment showed that the beam could withstand a load that was more than twice the design load and that the Chinese standard underestimated the actual bending capacity of the designed specimen.

## 3. Mock-Up Results and Discussion

### 3.1. Material

The mix design of UHPFRC is summarized in Table 1. As dispersed reinforcement, wires with a length of 60 mm and a diameter of 0.8 mm were used in an amount of 37 kg/m^3^. They were added to the mixture during mixing. Their distribution in the beam is omnidirectional and unoriented. The fiber volume fraction was 0.47%. A series of tests were performed to determine the material characteristics. The determination of the static modulus of elasticity was carried out based on the measurement of longitudinal deformations of test specimens during gradual compression loading, with the evaluation based on the linear part of the stress-strain curve in accordance with Hooke’s law. The tests were performed on cylindrical test specimens with a diameter of 150 mm and a height of 300 mm. The measurement of deformations was carried out using sensing devices mounted on the surface of the specimen in such a way as to minimize the effects of load eccentricity and local unevenness.

The loading was carried out in a controlled manner within a defined stress range, with repeated loading cycles, the aim of which was to stabilize the response of the material and eliminate the effect of initial nonlinearities. After the response stabilized, the static modulus of elasticity was determined from the ratio of the stress increment and the corresponding increase in relative strain between the lower and upper load levels. The static modulus of elasticity after 28 days was 46 GPa with a standard deviation of 1.2, while the bulk density of the concrete was 2419 kg/m^3^.

**Figure 4:**
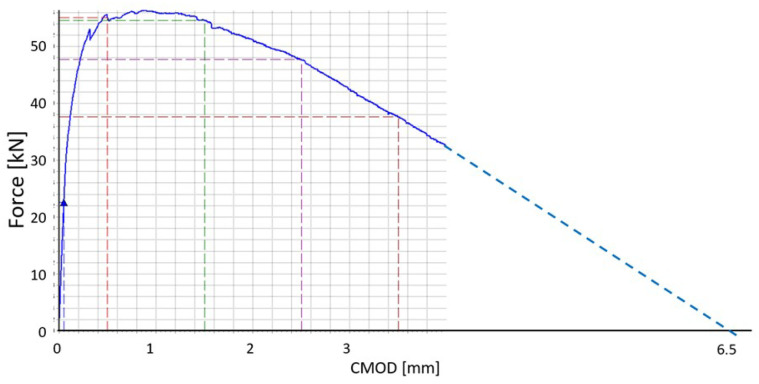
Force/CMOD diagram of three point bending beam with a notch.

A beam 150 × 150 × 700 mm with a notch was tested for softening at 28 days according to EN 14651+A1 [18]. Figure 4 shows the crack mouth opening displacement (CMOD) with regards to loading force. Assumption of linear softening resulted in CMOD = 6.5 mm. The proportional limit LOP $f_{\mathrm{ct},L}^{f}=7.0\;\mathrm{MPa}$ and the residual flexural tensile strength were then obtained at CMOD1 to CMOD4: $f_{r1,L}=17.2\;\mathrm{MPa}$, $f_{r2,L}=17.0\;\mathrm{MPa}$, $f_{r3,L}=14.9\;\mathrm{MPa}$, and $f_{r4,L}=11.7\;\mathrm{MPa}$.

Pressure tests were performed on cubes measuring 100 mm × 100 mm × 100 mm. The cubes were tested at ages of 7, 28, 56 and 90 days. All testing processes were carried out in accordance with the requirements of the ČSN EN 12390-3 standard [19]. The average strength after 7 days was 100.4 MPa, after 28 days 109.0 MPa, after 56 days 142.9 MPa, and after 90 days 144.6 MPa. The bulk density at 28 days was 2460 kg/m^3^ on water-cured samples.

Simultaneously with the pressure tests, tensile bending strength tests were performed on beams measuring 40 × 40 × 160 mm and 100 × 100 × 400 mm in accordance with the ČSN EN 12390-5 standard [20]. The tests were performed at an age of 28 days. In the first case, the strength was 25.6 MPa with a standard deviation of 1.7. In the second case, it was 18.9 MPa, with a standard deviation of 1.4. To verify the consistency of the measured values, compression tests were also performed on fragments remaining from the tensile bending tests. These were halves of beams loaded over areas of 100 × 100 mm and 40 × 40 mm. These fragments were tested at the age of 28 days. The compressive strength was 121.5 MPa with a standard deviation of 2.1 and 129.8 MPa with a standard deviation of 2.5.

The strength in uniaxial tension was tested on Dog Bone specimens aged 28 days and averaged 6.8 MPa with a standard deviation of 0.4.

### 3.2. Geometry

**Figure 5:**
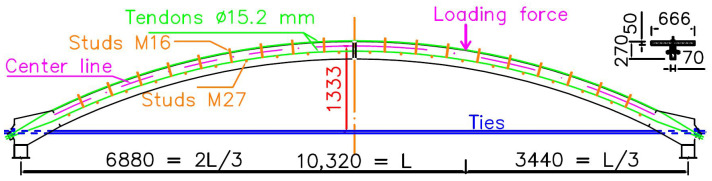
Longitudinal view and cross-section of the mock-up experiment (dimensions in mm).

A reduced model of the structure was chosen for the experiment at an approximate scale of 1:3; see Figure 5. The length of a single half-arch was 5.26 m, resulting in a total span of 10.32 m. The arch had a T-shaped cross-section with a flange thickness of 50 mm, a web width of 70 mm, and an overall cross-sectional depth of 270 mm. The mock-up geometry was designed to realistically represent the structural behavior of the full-scale ecoduct arch while remaining feasible for laboratory testing.

**Figure 6:**
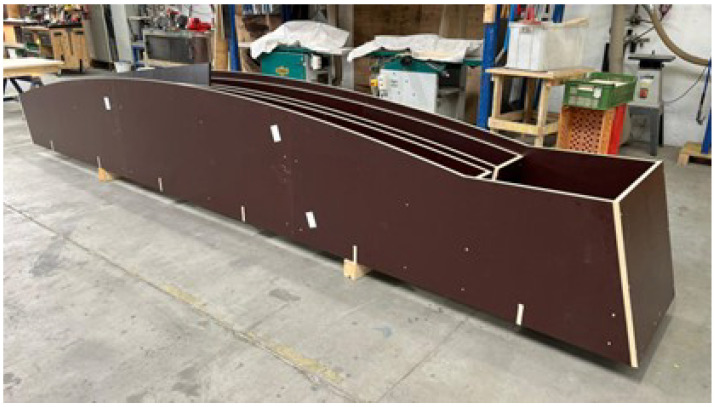
CNC-milled formwork assembled from smooth plywood panels for the casting of UHPFRC arch segments.

The choice of a 1:3 scale follows the widely accepted experimental practice in the field of concrete and composite structural testing. This scale has been used consistently in accordance with recommendations by RILEM and fib for the physical modelling of structural members, where it is essential to preserve adequate geometric similarity, realistic crack propagation, and a representative stress–strain state. A 1:3 scale is generally regarded as the smallest reduction that still maintains the correct interaction between bending, shear, and local instabilities, while avoiding the distortions that occur in significantly smaller models. Moreover, this scale effectively limits the size effect described in the fib Model Code 2010, ensuring that the mechanical response—particularly cracking behaviour, shear transfer mechanisms, and post peak softening—remains comparable to that of a full scale element. At the same time, a 1:3 model remains feasible for laboratory testing in terms of specimen weight, required loading capacity, and handling constraints, without compromising the fidelity of the results. For these reasons, the 1:3 scale is considered a standard and internationally recognized compromise between experimental accuracy and practical feasibility in the large-scale testing of concrete and UHPFRC structural components. Four identical formworks were fabricated for the production of UHPFRC beam segments. The formworks were manufactured from smooth formwork plywood panels, CNC-milled into individual components and subsequently assembled into the final curved geometry; see Figure 6. Prior to concreting, the formworks were positioned horizontally in an open area of the concrete plant to allow direct filling from a truck mixer. The ridge of the formwork was locally stiffened using M16 steel pins at a spacing of 500 mm. In addition, a drainage textile made of fine polypropylene fibers (Formtex) was bonded to the inner surface of the formwork. This textile enabled controlled drainage of excess air and water during casting, thereby locally reducing the water-to-cement ratio in the surface layer and leading to a denser concrete skin with reduced porosity. Openings were prepared in the formwork for the installation of steel guiding pins for the prestressing tendons in the web, together with ducts and anchorages for tendon routing and stressing.

**Figure 7:**
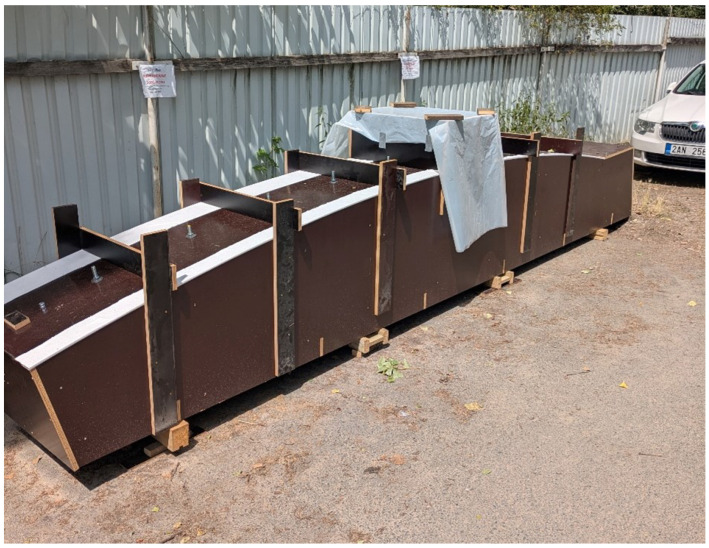
Casting of UHPFRC into the assembled formwork; filling was performed in stages and monitored through inspection and venting openings.

Concreting was performed in an upright position and through two casting openings. Initially, UHPFRC was poured into the formwork at the beam toe, where local reinforcement and anchorage elements for prestressing force transfer were located. After this section was completely filled, the lower opening was closed and concreting continued through a second opening positioned at the highest point of the formwork. The filling process was continuously monitored through inspection openings at the formwork crown, which simultaneously served as air vents; see Figure 7. After hardening, the beam segments were transported to the Experimental Centre laboratory of the Faculty of Civil Engineering, Czech Technical University in Prague. To ensure stability and protect the concrete elements during transport, demoulding was carried out only after the segments had been placed in the laboratory. Subsequently, M27 guiding pins were installed in the web at a spacing of 500 mm.

**Figure 8:**
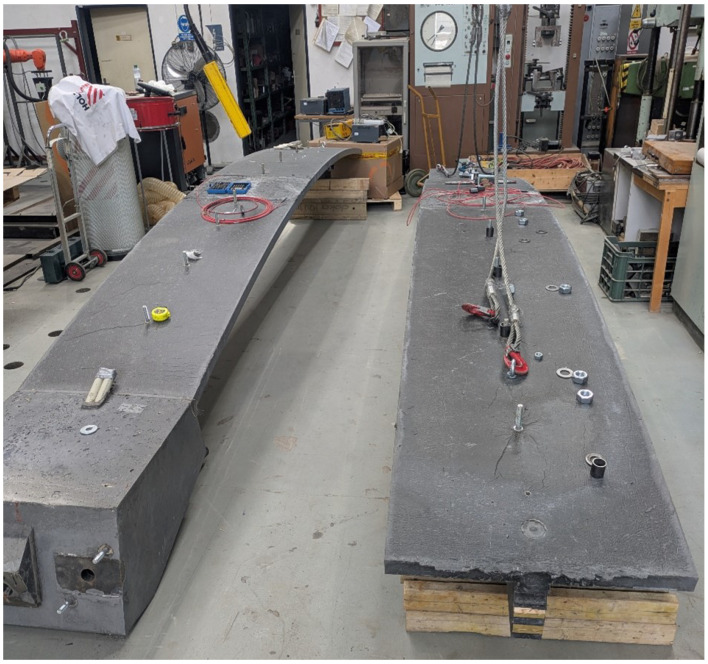
Assembly of two UHPFRC half-arch segments in mirror configuration prior to joint casting and post-tensioning.

The beam segments were then placed in a mirror configuration on elevated platforms to facilitate assembly and prestressing operations; see Figure 8. The opposing faces of the T-shaped profiles were shaped with negative shear keys to enable hinged interaction and accurate alignment of the two segments. A joint gap approximately 40 mm wide was formed, temporarily sealed with formwork, and filled with fresh UHPFRC. After curing the joint, both arches were post-tensioned using four external tendons with a diameter of 15.2 mm, anchored with VSL E-type anchorages and stressed to 1100 MPa. Compared to the real structure, the tendon layout was modified due to geometric constraints of the reduced cross-section. The diameter of tendon ducts could not be arbitrarily reduced without compromising the web integrity; therefore, the prestressing tendons were routed externally on both sides of the web. The prestressing force was transferred to the UHPFRC web by steel connectors installed at regular intervals of 500 mm.

The resulting uniform compressive stress in the arch reached −12.64 MPa, while the critical buckling stress was calculated as −67.0 MPa, indicating a sufficient safety margin against global instability. The primary objective of prestressing was to close potential hairline cracks at the joint and ensure watertightness of the assembled arch. Due to the curvature of the external tendons, a significant radial load of 57.7 kN/m was introduced, which considerably exceeded the expected uniform load from soil and vegetation in the real structure. Horizontal thrust forces were resisted by a pair of M36 threaded steel ties installed at the abutments.

The described geometry, production process, and prestressing arrangement defined the boundary conditions and internal force distribution of the experimental specimen.

### 3.3. Loading

**Figure 9:**
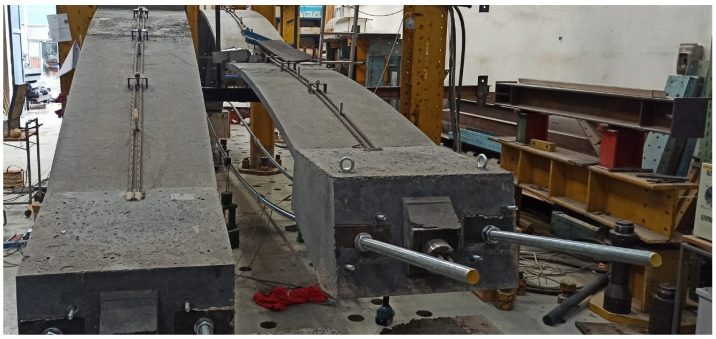
The soil load simulated by a pair of prestressed cables on the top of the beam.

Two types of loads can be assumed for an ecoduct. The first is from the layer of filled soil. This load will be along the entire length of the structure and will change within a certain range during the service life of the structure according to the drying and wetting of the soil. Arched structures withstand continuous loads very well and, given the relatively precise determination of the range in which this constant load will move, there is no problem in sizing the structure for it. The soil load was simulated using two prestressed ropes placed on the upper side of the beam. The ropes were placed symmetrically above the T-beam web; see Figure 9.

For arched beams, point loads, non-symmetrically placed, are much more dangerous. In the case of an ecoduct, these may be maintenance vehicles that mow grass or prune bushes, or vehicles that have nothing to do on the ecoduct, but their occurrence cannot be ruled out—agricultural or forestry equipment shortening their route, etc. This was simulated in the experiment by a point load, increasing until the beam collapsed. The first eigenshape of the arch is similar to point loading at 1/3 span and such a configuration was used for the mockup test. Four displacements were measured at 1/3 and 2/3 spans, and torsion effects were found negligible during testing. Four strain gages recorded strains at ties, and their mutual values were comparable. The displacement-controlled test was loaded using a hydraulic 3 MN cylinder with a maximum extension of 300 mm.

**Figure 10:**
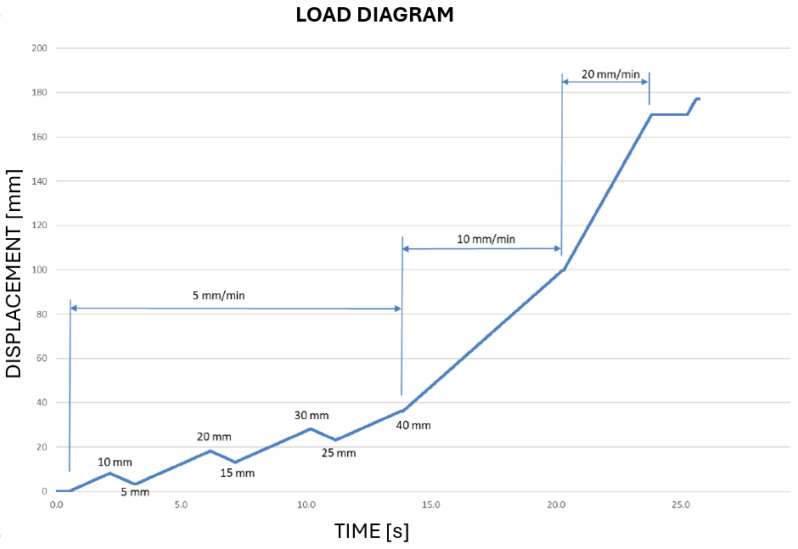
Loading diagram of *Experiment a*.

**Figure 11:**
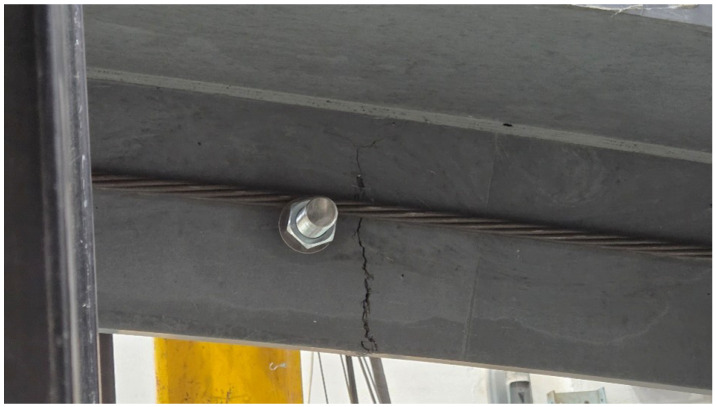
Development of tensile damage.

**Figure 12:**
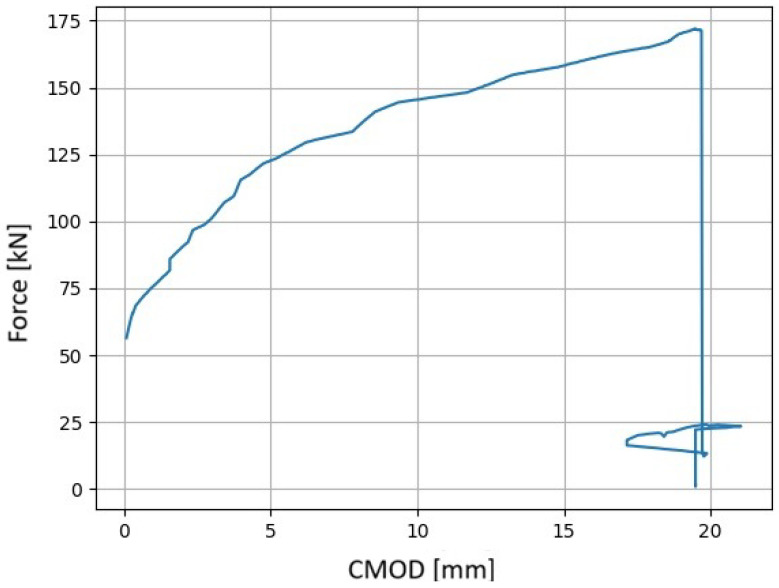
CMOD vs. loading force.

**Figure 13:**
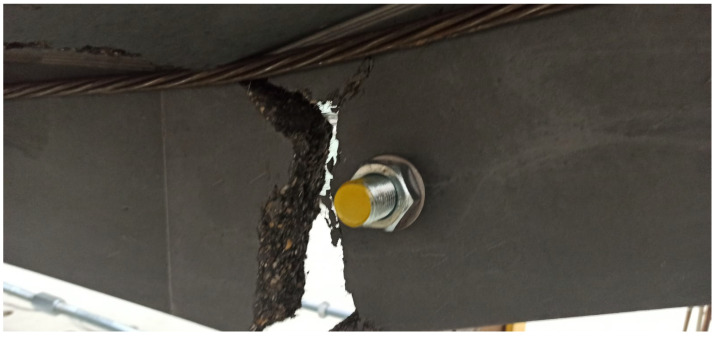
Experimental detail of first tensile crack.

**Figure 14:**
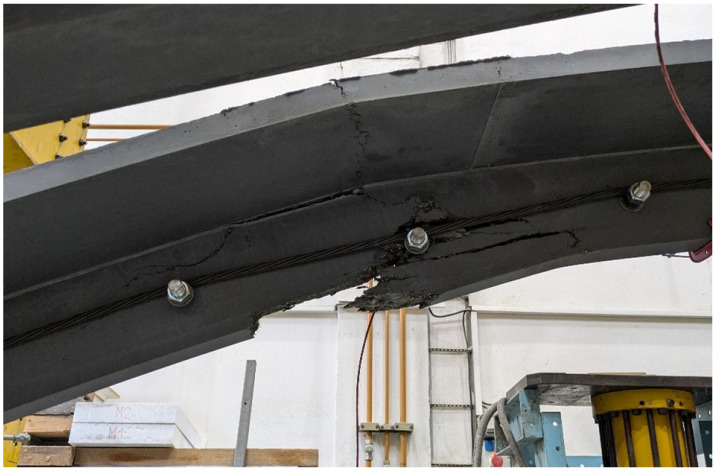
Experimental detail of second crack.

The loading of *Experiment a* took advantange of a few unloading steps; see Figure 10. The loading of *Experiment b* is similar, but without unloading steps. It followed only monotonous displacement. Figure 11 shows the initiation of the first tensile crack directly under the load. The crack width (CMOD) vs. applied load is shown in Figure 12. Figure 13 and Figure 14 show first and second crack after the beam reached its ultimate strength and collapsed.

**Figure 15:**
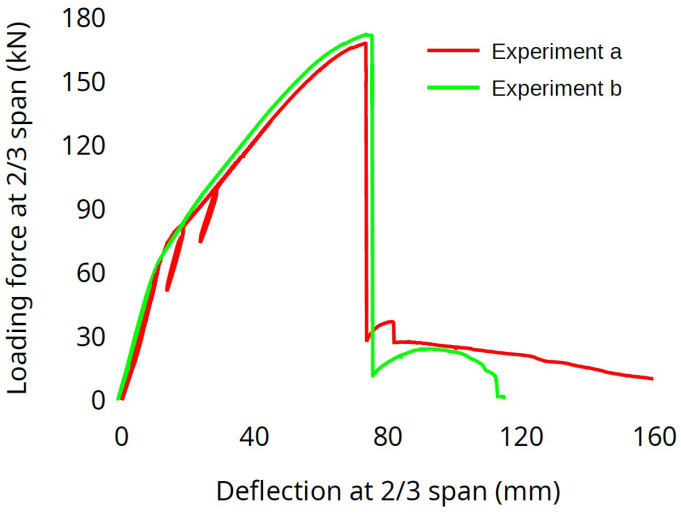
Force-displacement diagram from both experiments and the model.

Force-displacement diagrams in Figure 15 show that both arches behaved similarly.

Maximum loads from experiments yielded 167.9 and 172.0 kN. While the area under load is heavily damaged, the opposite cross section is still able to withstand high bending momentum.

**Figure 16:**
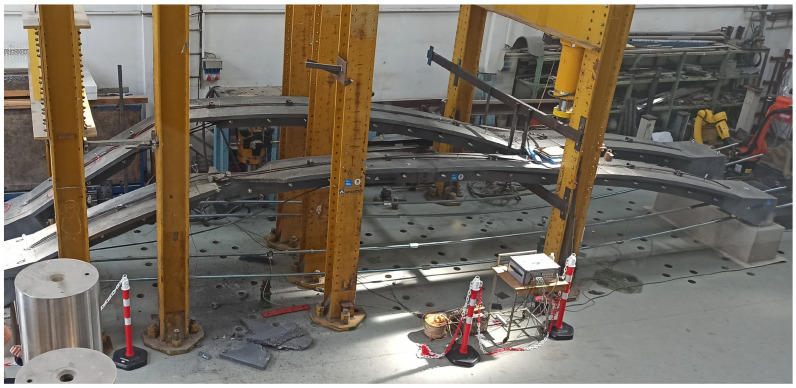
View of both broken arches.

Figure 16 shows the post-peak geometry when the arch is broken into three parts. Two hinges are created under the load and in the opposite cross-section. Significant compressive damage is located in the web. Indeed, *Experiment a* shot out a piece approximately 0.5 m long separated by shear inclined cracks. However, the web remained complete in *Experiment b*, showing only significant tensile cracking in both cross-sections.

**Figure 17:**
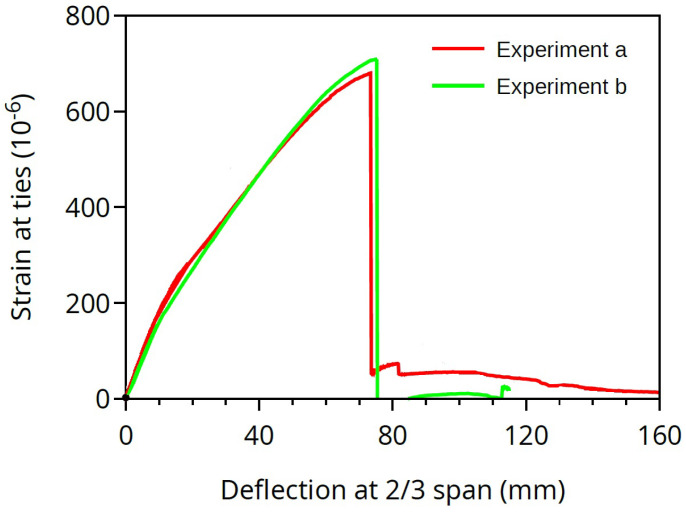
Strain at ties during loading.

Figure 17 demonstrates strain validation in ties, located at the bottom of the arch. The experiment shows lower strains due to partial restraint from foundation blocks. An overall view of both broken arch beams after the end of loading is shown in Figure 16.

## 4. Results and Discussion

- The experimental program was conducted on two specimens, which is consistent with established practice for the destructive testing of large-scale UHPFRC structural elements. For components of this size and complexity, the fabrication, handling, and testing of each additional specimen is associated with very high material, production, and loading frame costs, as well as significant logistical constraints. Both in the literature and in experimental mechanics practice, such limitations are recognized as a legitimate boundary condition for full-scale or near-full-scale testing of advanced cementitious composites.
- Crucially, both tested elements exhibited a consistent and repeatable failure mechanism, encompassing the tensile failure under the applied load and the compressive failure of the web. In the first specimen, a plastic hinge formed in the deck, allowing the prestressing to remain active until the end of the test. In the second specimen, local torsional cracking of the deck occurred, which can be attributed to a slight eccentricity in the applied load. These differences, however, do not represent distinct failure modes; rather, they constitute natural variations within the same fundamental failure mechanism, which is typical and expected in concrete structures.
- In both tests, the web failed in compression, the crack propagation pattern was comparable, and the global stress–strain response was nearly identical. From the perspective of experimental mechanics, the two specimens therefore provided consistent, reproducible, and mutually corroborating results, which is the primary criterion for the validity of destructive testing.
- International recommendations—such as those of RILEM Technical Committees and the fib Model Code—further emphasize that for large-scale structural elements, the quality and consistency of the observed failure mechanism is more important than the number of specimens. When the failure mechanism is reproduced across tests, the experiment is considered sufficiently demonstrative, even when only a small number of specimens can be tested.

## 5. Conclusions

The mock-up experiment successfully tested two arches made from UHPFRC. The conclusion can be summarized as follows:

- The model is reduced to approximately one third of the expected design. It can therefore be assumed that the effect of scale will be apparent. However, the scale of 1:3 can be considered relatively large and very accurate, and in bending stress, the scale effect for UHPFRC beams is generally almost non-existent [21].
- Casting and fabrication of arches is technologically possible, using high precision formwork. The T-shape of the slender arch allows optimal utilization of UHPFRC, taking advantage of watertightness, high compressive strength and distributed fiber reinforcement. The higher material price is significantly reduced by decreased consumption.
- The mock-up experiment presents approximately a 1:3 scale of a real ecoduct. No significant issues were found for upscaling, opening new material methods for traditional arch bridges.

## Figures and Tables

**Table 1:** Composition of the UHPFRC mixture (NSC1).

Component	Dosage (kg/m^3^)
Portland clinker	550
CO_2_ from clinker	396
Microsilica	yes
Water	72
Aggregates	1785.0
Microfibres	37

## Data Availability

The original contributions presented in this study are included in the article material. Further inquiries can be directed to the corresponding authors.

## Abbreviations

The following abbreviations are used in this manuscript:

UHPFRC	Ultra-high-performance fiber-reinforced concrete
UHPC	Ultra-high-performance concrete
SCC	Self-compacting concrete
FRC	Fiber-reinforced concrete
HPC	High-performance concrete
NC	Normal concrete
CMOD	Crack mouth opening displacement
MSWIFA	Municipal solid waste incineration fly ash
